# Facilitation or inhibition? Impact of CEO’s financial background on industrial AI transformation of manufacturing companies

**DOI:** 10.3389/fpsyg.2023.1126801

**Published:** 2023-03-02

**Authors:** Peng Xu, Zichao Zhang

**Affiliations:** ^1^School of Business Administration and Corporate Governance Research Center of Shandong University of Finance and Economics, Jinan, China; ^2^School of Business Administration, Shandong University of Finance and Economics, Jinan, China

**Keywords:** financial background, industrial AI transformation, corporate governance, parent-subsidiary corporation, enterprise financialization

## Abstract

**Introduction:**

The purpose of this paper is to empirically test the impact of CEO’s financial background on industrial AI transformation of manufacturing enterprises based on upper echelons theory and imprinting theory.

**Methods:**

The paper preliminarily takes listed manufacturing companies in Shanghai and Shenzhen stock markets that are affiliated to enterprise groups from 2014 to 2020 as samples, and manually collects and collates datas of CEO’s financial background and industrial AI transformation. The research hypotheses are tested by stata 15.0 software.

**Results:**

It is found that CEO’s financial background significantly inhibits the industrial AI transformation of manufacturing enterprises, and when the CEO works part-time in the parent company, it will strengthen the negative impact of CEO’s financial background on industrial AI transformation. Further research shows that enterprise financialization plays a partial intermediary role between CEO’s financial background and industrial AI transformation; Compared with private enterprise groups, the inhibiting effect of CEO financial background on industrial AI transformation is stronger in state-owned enterprise groups; CEOs with non-banking financial background have a stronger inhibitory effect on industrial AI transformation.

**Discussion:**

Firstly, based on the process of making business decisions, it verifies and clarifies the action mechanism of CEO’s financial background on industrial AI transformation through internal driving mechanism, which expands the research horizon of industrial AI transformation, and further applies the Imprinting Theory in biology to the research of business decision-making, which forms a beneficial complement to the relevant research on economic consequences of CEO’s financial background. Secondly, different from the research of single independent company, this paper focuses on the special situation of parent-subsidiary corporate governance, and explores the mechanism of action, deepening the research on the synergy of enterprise groups. Finally, this paper further explores the influence of CEO’s financial background on industrial AI transformation, which is conducive to a deeper understanding of the heterogeneity of managers except manpower and capital factors in the industrial AI transformation practice of manufacturing enterprises, and provides a new idea and a more comprehensive analysis perspective for industrial AI transformation.

## 1. Introduction

Under the background of the increasing downward pressure of the current macroeconomy, the comparative advantage of traditional manufacturing industry in international competition begins to weaken, and the problem of “large but not strong, comprehensive but not excellent” in manufacturing industry becomes more and more prominent (Li et al., 2017). At the same time, most developed countries have taken intelligent manufacturing as a major strategy to plan the layout and take active measures to seize the initiative of industrial competition (Zhong et al., 2017; Kuo et al., 2019; Osterrieder et al., 2020). How to explore the transformation route and breakthrough point has become an issue of the times that Chinese manufacturing enterprises must answer. Since the 19th National Congress of the CPC, governments at all levels have attached great importance to the application of digital technologies such as artificial intelligence (AI) in order to adapt to the global innovation of manufacturing technology. In April 2021, MIIT issued the “14th Five-Year Plan for Intelligent Manufacturing Development (Draft for Comment),” which called for the fundamental transformation of manufacturing industry model and enterprise form. In 2022, the Government Work Report pointed out that “promoting the upgrading of traditional industries and vigorously promoting intelligent manufacturing,” which clarified the strategic positioning of artificial intelligence application in the top-level design.

As the main direction of “Made in China 2025,” intelligent manufacturing is in line with the inherent requirements of the development direction of manufacturing enterprises (Tsang and Lee, 2022), and is conducive to reshaping the competitive advantage in manufacturing enterprises and promoting the transformation and upgrading of industrial structure (Marques et al., 2017; Lee et al., 2022). The depth coupling of artificial intelligence and the substantial economy has become top priority to the high-quality development of manufacturing enterprises, and it is also an important guarantee for implementing the strategy of “manufacturing power” and building a domestic and international dual-cycle system (He and Bai, 2021). In this context, it is very necessary to study the antecedents affecting the industrial AI transformation of manufacturing enterprises from the internal perspective at the micro level.

As China’s capital market steps into a new stage, financial activities are in a good situation, and it has become a unique phenomenon in the process of China’s economic reform that more and more financial practitioners are employed as CEOs in enterprises (Ying and He, 2020). Due to the great difference between the financial activities as a virtual economy and the operation and management of the real economy, financial experience may leave deep memories and habitual thinking for executives, which will have a lasting and far-reaching impact on the personality characteristics and behavior patterns of corporate CEOs, and finally reflect on corporate behavior decisions (Hermano and Martin-Cruz, 2016; Mun et al., 2020). Previous studies have examined various economic consequences of CEO’s financial backgrounds, including internal control weaknesses (Oradi et al., 2020), earnings management (Gounopoulos and Pham, 2018), timeliness of audit reports (Salehi et al., 2018) and corporate innovation (Yang et al., 2021). Under the policy background of deepening the popularization and application of intelligent manufacturing in China, whether the CEO of manufacturing enterprises should be a person with financial experience, and whether the financial background of CEOs is at odds with industrial AI transformation which avoids the tendency of “turning reality into emptiness,” the relevant research is still lacking at present. It is urgent to open the “black box” between the financial background of CEOs and industrial AI transformation.

This study focuses on the following questions: What is the impact of CEO’s financial background on industrial AI transformation? And what is the mechanism of its impact? Further, enterprise groups composed of numerous subsidiaries play pivotal roles in economic growth, while listed subsidiaries, as subsystems embedded in enterprise groups, can realize resource sharing within enterprise groups (Dou et al., 2021; Min et al., 2022; Zheng et al., 2022). What are the differences in the performance of listed subsidiaries in enterprise groups in developing and applying AI as opposed to independent or single companies.

Based on the above considerations, this article is sampled by the listed manufacturing companies belonging to enterprise groups in Shanghai and Shenzhen stock markets from 2014 to 2020, and empirically examines the impact of CEO’s financial background on industrial AI transformation and the contingency situation in the action path. The study makes the following possible contribution. Firstly, based on the process of making business decisions, it verifies and clarifies the action mechanism of CEO’s financial background on industrial AI transformation through internal driving mechanism, which expands the research horizon of industrial AI transformation, and further applies the Imprinting Theory in biology to the research of business decision-making, which forms a beneficial complement to the relevant research on economic consequences of CEO’s financial background. Secondly, different from the research of single independent company, this paper focuses on the special situation of parent-subsidiary corporate governance, and explores the mechanism of action, deepening the research on the synergy of enterprise groups. Finally, this paper further explores the influence of CEO’s financial background on industrial AI transformation, which is conducive to a deeper understanding of the heterogeneity of managers except manpower and capital factors in the industrial AI transformation practice of manufacturing enterprises, and provides a new idea and a more comprehensive analysis perspective for industrial AI transformation.

## 2. Theoretical analysis and hypothesis development

### 2.1. Theoretical background

In this study, we use upper echelons theory and imprinting theory as the theoretical framework. According to the upper echelons theory, enterprise decision-making is essentially the result of environmental factors being filtered and selected by executives’ bounded rationality, and executives’ cognitive foundation and values are the key factors determining enterprise decision-making (Hambrick and Mason, 1984), and previous studies have applied upper echelons theory to analyze the effects of different types of CEO on business decision (Bernile et al., 2017; Sunder et al., 2017). According to the imprinting theory, CEO’s management skills are not innate, and their professional experience will leave a deep impression on their psychology (Mathias et al., 2015), which greatly affects their cognitive structure, value orientation and decision-making mode (Benmelech and Frydman, 2015; Schoar and Zuo, 2017). Work in the financial field is full of challenges and pressures, and the deep memories formed will have a significant impact on their future decision-making orientation (Chao et al., 2017). Based on this, as the helm of the enterprise, the CEO is responsible for the decision-making of the enterprise, and his financial background will inevitably affect the behavior decision of the enterprise by influencing his personality characteristics.

### 2.2. CEO’s financial background and industrial AI transformation

This paper holds that the CEO with financial background is more motivated and capable to restrain the industrial AI transformation of manufacturing enterprises. On the one hand, the CEO’s personality characteristics shaped by the work experience in the financial industry may repel the industrial AI transformation. China’s capital market has a relatively short history of development, and the investment concept is not yet mature (Huang and He, 2020; Yang et al., 2021). Finance is an industry that faces many temptations and great pressure. Growing and working in such a capital market environment, they are likely to be affected by speculative thinking (Yurttadur and Ozcelik, 2019), thus developing their personal traits of preference for speculation and stronger profit-seeking motivation. The industrial AI transformation of manufacturing enterprises is not a simple one-time investment, but an investment process with long cycle, high risk and strong uncertainty, which has a strong state dependence (Anderson et al., 2022). In contrast, financial investment has a shorter return cycle and a higher rate of return. Therefore, CEOs with financial background may have short-sighted behavior out of the motivation of quick success or risk avoidance, and prefer to invest limited funds in financial market (Liu et al., 2020). Moreover, based on the system of separation of two rights, two kinds of agency problems make CEO tend to give priority to shareholder value when deciding the order of maximizing the operating profit of the enterprise and maximizing the interests of shareholders due to their principal-agent responsibilities (Kiefer et al., 2017), which makes enterprises more willing to invest in high-yield financial assets. It will lead to the reduction of investment in industrial AI transformation, and eventually distort the industrial AI transformation and upgrading of entity enterprises.

On the other hand, CEOs with financial background may cause a “crowding-out effect” on industrial AI transformation. First, financial investment is familiar field and “comfort zone” for CEOs with financial background (Custodio and Metzger, 2014). The advantages of CEO’s financing convenience can easily lead to excessive deviation of corporate capital structure and increase the expansion of inefficient investment of enterprises (Zhang, 2022). Moreover, CEOs with financial background have a relatively poor understanding of the cutting-edge technology and development direction of artificial intelligence in their working career, and it is difficult to provide efficient guidance and forward-looking decision-making for industrial AI transformation of manufacturing enterprises, thus resulting in invalid or inefficient R&D resource allocation, which is obstruct to the smooth implementation of industrial AI transformation projects. Second, when the CEO with financial background gains profits through financial investment, the short-term profit may further aggravate the CEO’s short-term investment tendency, shorten the planning horizon of the company’s management, thus forming the “path dependence” on the financial profit channel (Wang et al., 2021), which eventually leads to the weakening of the motivation of continuous technological improvement, and makes it difficult to sustain the transformation and upgrading of enterprises.

In summary, the following hypothesis is proposed:

*Hypothesis 1*: The CEO’s financial background has a significant negative impact on the industrial AI transformation of manufacturing enterprises.

### 2.3. The moderating effect of the part-time job of the subsidiary CEO in the parent company

Under the specific ownership arrangement and governance structure of listed companies in China, there is a common phenomenon in enterprise groups that the CEO holds a part-time job in the parent company (Opie et al., 2019). This paper argues that the part-time job of the subsidiary CEO in the parent company can strengthen the negative impact of the CEO’s financial background on the industrial AI transformation. The specific logic is as follows:

First, CEO’s presence in the parent company can effectively alleviate the information asymmetry and weakened control, so that the parent company can achieve a certain degree of direct control over listed companies at the executive level (Cai et al., 2019), making investment decisions more in line with the judgment of the parent company. Under strict vertical control and compliance supervision, the CEO will adopt management strategies closer to the parent company’s preferences (Freund et al., 2021), further shortening the CEO’s vision of investment planning. The innovation enthusiasm of CEO who prefers speculation is greatly weakened, and prefer to invest in high-yielding financial assets, which hinders industrial AI transformation of entity enterprises. Second, CEO’s part-time in the parent company is a manifestation of power enhancement. The dual-attribute CEO is prone to overconfidence because of more discourse power and a higher degree of control over social resources. In this case, CEO prefers his own confident financial investment and strengthens the circular path of financial investment, which makes a crowding out effect on the application of artificial intelligence, thus inhibiting the implementation and execution of industrial AI transformation.

In summary, the following hypothesis is proposed:

*Hypothesis 2*: When the CEO of the subsidiary works part-time in the parent company, the financial background of the CEO has a stronger inhibitory effect on the subsidiary’s industrial AI transformation.

**Figure 1:**
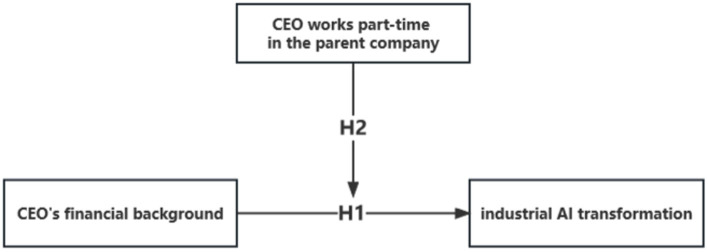
Research framework.

Based on the above analysis, the research framework constructed in this paper is shown in Figure 1.

## 3. Methodology and variable definitions

### 3.1. Sample selection and data source

Reference to the research of Xu et al. (2021), this article makes a preliminary sample selection of A-share manufacturing companies listed in Shanghai and Shenzhen stock markets by referring to the company control chain diagram and annual report, selects the listed subsidiaries of enterprise groups as the initial sample, and limits the sample observation interval to “2014–2020.” Drawing on existing studies, this paper adopts the following criteria for sample selection: (1) exclude financial listed companies; (2) exclude ST, *ST and listed companies that were delisted during the observation period; (3) eliminate listed companies with missing main variables. To eliminate the impact of extremum on the research conclusions, all continuous-type variables are processed by winsorize at 1 and 99% levels, and 4,852 observation samples are finally obtained. The industrial AI transformation data is collected manually from annual reports of listed companies for the period 2014–2020, and other main variables and control variables are obtained from the CSMAR database.

### 3.2. Variable definitions

#### 3.2.1. Industrial AI transformation

**Figure 2:**
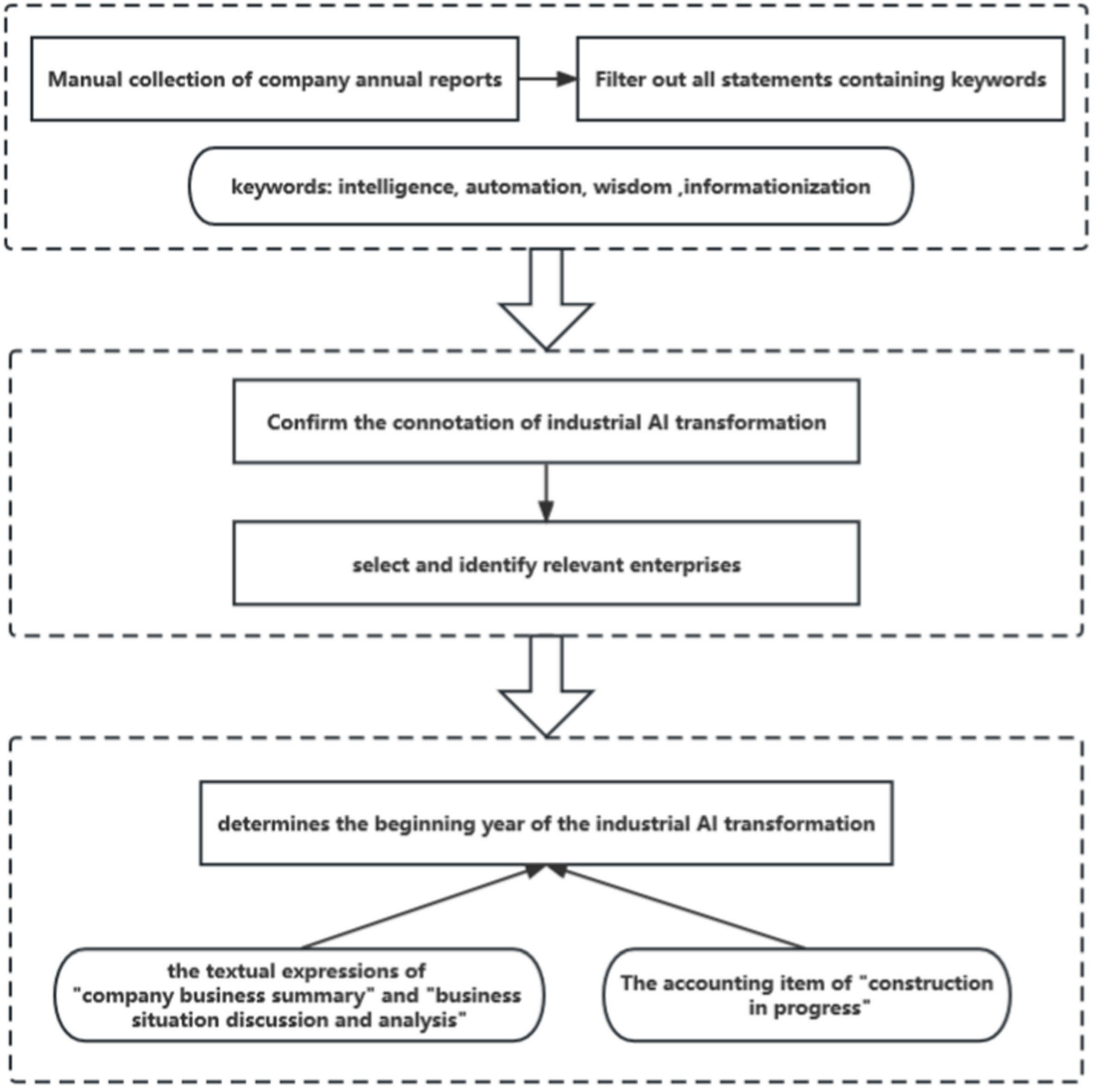
The measuring method of industrial AI transformation.

The measuring method of industrial AI transformation is shown in Figure 2. This paper adopts the double difference method (DID) to construct the measurement index AI*YEAR. Firstly, the dummy variable AI it is constructed, indicating whether company i has undergone industrial AI transformation, and the industrial AI transformation enterprise is 1, otherwise, it is assigned to 0. Then, the dummy variable YEAR is constructed to indicate the year that i company has undergone industrial AI transformation, and the implementation year is 1, otherwise it is 0. The specific steps are as follows: Firstly, annual reports of all sample companie from 2014 to 2020 were collected manually, and words reflecting industrial AI transformation such as “intelligence,” “automation,” “wisdom,” and “informationization” were selected to filter out all statements containing keywords; Secondly, based on the connotation of industrial AI transformation, enterprises that conform to the deep integration of the new generation of information technology and manufacturing are selected and identified as industrial AI transformation enterprises, with AI as 1; Finally, this paper manually determines the beginning year of the industrial AI transformation from the following two aspects: (1) The year in which the enterprise applied artificial intelligence products is involved in the textual expressions of “company business summary” and “business situation discussion and analysis.” For example, Shenzhen Zhongheng Huafa Co., Ltd. has updated some old injection molding machine equipment in 2014, and the energy-saving effect has continued to appear. In addition, with the implementation of automation improvement and process optimization process, the waste of manpower input and production materials has greatly reduced, and the production efficiency has been significantly improved; (2) The accounting item of “construction in progress” refers to the year when the project applied by “artificial intelligence” has been completed and has reached the expected state of use. For example, Shenzhen Danbang Technology Co., Ltd. completed the project of intelligent monitoring system for the whole process of sewage discharge in 2020 and started operation. Finally, the measurement index AI*YEAR of industrial AI transformation variables is obtained.

#### 3.2.2. CEO’s financial background

Referring to previous studies, CEOs with financial background are defined as those who have worked in financial regulatory authorities, policy banks, commercial banks, insurance companies, securities companies, fund management companies, securities registration and settlement companies, futures companies, investment banks, trust companies, investment management companies, exchanges and other financial companies. The CEO with financial background is assigned a value of 1, and the CEO without financial background is assigned a value of 0. The data of this variable comes from the CSMAR Database of Chinese Listed Companies.

#### 3.2.3. The CEO works part-time in the parent company

When the CEO works part-time in the parent company, the value is assigned as 1, and when the CEO does not work part-time in the parent company, the value is 0. The data of this variable also comes from CSMAR database of character traits of Chinese listed companies.

#### 3.2.4. Control variables

Refer to previous research, this paper controls the following in regression analysis: the ownership concentration (TOP1), the asset-liability ratio (LEV), the board size (BOD), the proportion of independent directors (INDE), operating cash flow (CASH), the return on assets (ROA), the company age (AGE). In addition, Year denotes year fixed effect and Industry denotes industry fixed effect. The definition and measurement of variables are shown in Table 1.

**Table 1:** Variable definitions.

Code	Variables	Index
INM	Industrial AI transformation	Indicates whether the enterprise has undergone industrial AI transformation. See the formula above for the specific measurement method.
FC	CEO’s financial background	The CEO with financial background is assigned as “1”; Otherwise, “0.”
ET	The CEO works part-time in the parent company	The CEO works part-time in the parent company, is assigned as “1”; Otherwise, “0.”
TOP1	The shareholding ratio of the largest shareholder	The proportion of shares held by the largest shareholder of the listed company to the total share capital.
LEV	The asset-liability ratio	The year-end asset-liability ratio of listed companies.
BOD	The board size	The number of board members of listed companies
INDE	The proportion of independent directors	The proportion of independent directors to the total board of directors of listed companies.
CASH	Operating cash flow	The ratio of annual net operating cash flow of listed companies to total assets at the end of the period.
ROA	The return on assets	The ratio of net profit to total assets of listed companies.
AGE	The company age	The number of years the listed company has been listed.
YEAR	Year	Dummy variable, the year of the observation sample belongs to this year and is recorded as “1,” otherwise it is “0.”
INDUSTRY	Industry	classification standards of the China Securities Regulatory Commission.

### 3.3. Models

To test the hypothesis of this paper, the following regression models are designed for this study. Model (1) is used to test the effect of CEO’s financial background on industrial AI transformation, and model (2) is used to test the moderating role of CEO works part-time in the parent company between CEO’s financial background and industrial AI transformation.


$$INM=c+\alpha_{1}FC+\overset{n}{\underset{j=1}{\sum }}\left(b_{j}Control\right)+\varepsilon$$

$$INM=c+\alpha_{1}FC+\alpha_{2}ET+\alpha_{3}FC*ET+\overset{n}{\underset{j=1}{\sum }}\left(b_{j}Control\right)+\varepsilon$$

Among them, FC*ET denotes the interaction term of CEO’s financial background and the dummy variable of the CEO works part-time in the parent company, *Control* is the control variable described previously; *c* is the intercept term; ε represents the error perturbation term, *j* is the number of each control variable, *bj* represents the regression coefficient of each control variable, and *α* represents the regression coefficient of the explanatory variables.

## 4. Data analysis and results discussion

### 4.1. Descriptive statistics

Table 2 reports the results of descriptive statistical of the main variables in the models. As can be seen from Table 2, the mean and standard deviation of industrial AI transformation (INM) are 0.466 and 0.499, respectively, indicating that there are still many listed subsidiaries that have not yet carried out industrial AI transformation; The mean of CEO’s financial background (FC) is 0.042, indicating that CEOs with financial background in the sample accounts for only 4.2%, and most of the CEOs of listed companies have no financial background; The statistical characteristics of the remaining control variables are basically close to existing research literature, so will not be repeated here.

**Table 2:** Descriptive statistics.

Variables	Number	Minimum	Median	Maximum	Mean	Standard Deviation
INM	4,852	0.000	0.000	1.000	0.466	0.499
FC	4,852	0.000	0.000	1.000	0.042	0.202
ET	4,852	0.000	0.000	1.000	0.358	0.479
TOP1	4,852	0.111	0.341	0.740	0.356	0.135
LEV	4,852	0.076	0.436	0.908	0.443	0.187
BOD	4,852	5.000	9.000	14.000	8.746	1.540
INDE	4,852	0.333	0.333	0.571	0.370	0.052
CASH	4,852	−0.128	0.044	0.222	0.047	0.060
ROA	4,852	−0.242	0.031	0.198	0.034	0.061
AGE	4,852	1.386	2.773	3.296	2.600	0.506

### 4.2. Multiple collinearity test

In order to ensure that the regression results will not be biased due to multicollinearity, this paper carries out variance inflation factor test on explanatory variables and control variables. According to the judgment criterion of VIF value of variance inflation coefficient, 10 is usually taken as the critical value. When variance inflation coefficient is less than 10, it can be judged that the multiple regression models do not have serious multicollinearity problem, the closer the value of variance inflation coefficient is to 1, the lighter the collinearity problem in multiple regression models, and the better the regression results of the models. It can be seen from Table 3 that the maximum VIF value is 1.33, which is far less than 10. Thus there is basically no multicollinearity between explanatory and control variables, and regression analysis of causality between variables can be carried out.

**Table 3:** Multiple collinearity test.

Variables	VIF	1/VIF
FC	1.01	0.99
TOP1	1.07	0.94
LEV	1.24	0.81
BOD	1.27	0.79
INDE	1.23	0.81
CASH	1.20	0.83
ROA	1.33	0.75
AGE	1.09	0.92

### 4.3. Multiple regression results

To verify the research hypotheses proposed above, it is tested by stata15 software. Table 4 shows the specific regression analysis results of CEO’s financial background on industrial AI transformation. Column (1) shows the result of regression analysis without control variables. It can be seen that the regression coefficient of CEO’s financial background (FC) is −0.395, which is significant at 5% level. The results of column (2) after adding control variables show that the regression coefficient of CEO’s financial background (FC) is −0.360, which is significant at 5% level. All the above results show that there is a significant negative relationship between CEO’s financial background and industrial AI transformation, that is, CEO’s financial background is not conducive to industrial AI transformation of manufacturing enterprises. The hypothesis H1 in the previous research has been verified. In column (3), the coefficient of the product term FC*ET between the CEO’s financial background (FC) and CEO’s part-time job in the parent company (ET) is 0.932, which is significant at the level of 1%, indicating that the negative impact of the CEO’s financial background on industrial AI transformation will be strengthened when the CEO works part-time in the parent company. Hypothesis H2 of the previous study is verified.

**Table 4:** Multiple regression analysis results.

Variables	INM (1)	INM (2)	INM (3)
FC	−0.395** (−2.55)	−0.360** (−2.30)	−0.735*** (−3.52)
ET			0.256*** (3.75)
FC*ET			0.932*** (2.83)
TOP1		−0.080 (−0.32)	−0.125 (−0.50)
LEV		1.253*** (6.31)	1.196*** (6.00)
BOD		0.079*** (3.39)	0.077*** (3.26)
INDE		2.544*** (3.77)	2.319*** (3.42)
CASH		1.861*** (3.17)	1.813*** (3.09)
ROA		2.776*** (4.55)	2.652*** (4.33)
AGE		−0.483*** (−6.76)	−0.432*** (−5.97)
Year	Yes	Yes	Yes
Industry	Yes	Yes	Yes
Constant term	−0.607*** (−2.80)	−1.849*** (−3.88)	−1.873*** (−3.91)
N	4,852	4,852	4,852
R^2^	0.089	0.108	0.112
F	599.20	721.56	749.26

The value of *t* is in parentheses.

*** Means *p* < 0.01.

** Means *p* < 0.05.

* Means *p* < 0.1.

## 5. Robustness

### 5.1. Propensity score matching

To ensure the robustness of the study results and to solve the problem of sample self-selection, this paper uses the propensity score matching method (PSM) to match a sample of companies with industrial AI transformation in a 1:1 neighborhood. The model variables for calculating propensity scores include TOP1, LEV, BOD, INDE, CASH, ROA, and SIZE, and the Logit model was used for regression analysis of the matched sample data. Column (1) of Table 5 reports the regression results. The regression coefficient of CEO’s financial background (FC) is −0.707, which is significant at the level of 10%, indicating that previous conclusion is still valid.

**Table 5:** Robustness.

Variables	INM (1)	INM (2)	INM (3)
FC	−0.707* (−1.65)		−0.217** (−2.28)
L.FC		−0.310* (−1.80)	
TOP1	−0.785 (−0.85)	−0.121 (−0.44)	−0.050 (−0.33)
LEV	−1.374 (−1.47)	1.400*** (6.46)	0.754*** (6.29)
BOD	0.065 (0.67)	0.082*** (3.19)	0.049*** (3.45)
INDE	2.462 (0.79)	2.638*** (3.63)	1.572*** (3.83)
CASH	−1.911 (−0.62)	2.580*** (4.07)	1.080*** (3.05)
ROA	0.098 (0.05)	2.685*** (4.11)	1.696*** (4.61)
AGE		−0.496*** (−6.18)	−0.295*** (−6.83)
SIZE	0.342*** (2.77)		
Year	Yes	Yes	Yes
Industry	Yes	Yes	Yes
Constant term	−9.405*** (−3.30)	−1.604*** (−3.10)	−1.126*** (−3.88)
N	399	3,992	4,852
R^2^	0.171	0.087	0.108
F	93.85	480.94	721.43

The value of *t* is in parentheses.

*** Means *p* < 0.01.

** Means *p* < 0.05.

* Means *p* < 0.1.

### 5.2. Other robustness checks

To ensure the reliability of the research conclusions, we also carried out the following robustness tests: (1) lag variables. The exploration process and achievement of industrial AI transformation are relatively long, and the CEO with financial background are likely to reap the achievements of their predecessors in industrial AI transformation. In order to avoid the endogenous deviation caused by “predecessors planting trees, later generations enjoying the cool,” this paper will lag the explanatory variables by one period, and use Logit model to estimate. Column (2) in Table 5 reports the regression result, which is still consistent with the hypothesis in this paper. (2) Change the measurement method. Change the test model of the impact of CEO’s financial background on industrial AI transformation, and re-test with Probit model. Column (3) in Table 5 shows the specific regression results, which are consistent with the previous conclusions.

## 6. Further analysis

The regression results above prove that CEO’s financial background inhibits the industrial AI transformation of manufacturing enterprises, but its internal mechanism still needs to be tested. Based on this, this part will explore the influence mechanism between the two, and introduce the property right nature of enterprise groups and the heterogeneity of CEO’s financial background types into the research framework to further analyze the influence of CEO’s financial background on industrial AI transformation.

### 6.1. Intermediary mechanism test of enterprise financialization

The financial work experience has left a deep “mark” on CEO, which makes the CEO’s decision-making more inclined to financial investment; At the same time, they use their own “circle” to bring social capital to enterprises, effectively alleviate financing constraints and reduce capital possession, which has increased the degree of enterprise financialization (Shi et al., 2021). Enterprises’ financial asset allocation is more motivated by “profit-seeking,” and they prefer to engage in short-term speculative financial investment (Jin et al., 2022), thus hindering the transformation of industrial AI in manufacturing enterprises. To verify the influence mechanism of CEO’s financial background on industrial AI transformation, the following models are constructed for regression test:


$$FINRATIO=c+\alpha_{1}FC+\overset{n}{\underset{j=1}{\sum }}\left(b_{j}Control\right)+\varepsilon$$

$$INM=c+\alpha_{1}FINRATIO+\alpha_{2}FC+\overset{n}{\underset{j=1}{\sum }}\left(b_{j}Control\right)+\varepsilon$$

Among them, FINRATIO is the intermediary variable, which indicates the degree of enterprise financialization. Specifically, this paper adopts the ratio of financial assets to total assets to define enterprise financialization. Table 6 reports the test results of enterprise financialization as an intermediary mechanism. According to the regression results of model (3), there is a significantly positive correlation between CEO’s financial background (FC) and enterprise financialization (FINRATIO) at the level of 1%, indicating that CEO’s financial background reinforces the trend of enterprise financialization. According to the regression result of model (4), the regression result of CEO’s financial background (FC) is significantly negative. In addition, the regression result of enterprise financialization (FINRATIO) is significant at the level of 5%, which means that enterprise financialization has a significant negative impact on industrial AI transformation. The above results show that CEO’s financial background can inhibit industrial AI transformation of enterprises by improving the path of enterprise financialization, that is, enterprise financialization plays a significant part of intermediary role between CEO’s financial background and industrial AI transformation, which is in line with the expected assumption of this paper.

**Table 6:** Further analysis.

Variables	FINRATIO (1)	INM (2)	INM (3)	INM (4)	INM (5)
FC	0.018*** (4.50)	−0.335** (−2.13)	−0.102 (−0.52)	−0.732** (−2.52)	
FINRATIO		−1.459** (−2.50)			
BFC					−0.429 (−1.24)
NBFC					−0.375** (−2.02)
Controls	Yes	Yes	Yes	Yes	Yes
Year	Yes	Yes	Yes	Yes	Yes
Industry	Yes	Yes	Yes	Yes	Yes
Constant term	0.032*** (2.64)	−1.808*** (−3.79)	−1.110 (−1.47)	−3.426*** (−4.48)	−1.843*** (−3.87)
N	4,852	4,852	2,484	2,353	4,852
R^2^	0.070	0.109	0.101	0.151	0.108
F	9.680	727.89	345.80	490.25	721.86

The value of *t* is in parentheses.

*** Means *p* < 0.01.

** Means *p* < 0.05.

* Means *p* < 0.1.

### 6.2. Heterogeneity analysis of property rights

Many previous studies have confirmed that in China’s institutional environment, the internal governance logic and decision-making mechanisms of state-owned enterprises and private enterprises are significantly heterogeneous (Clarke, 2003). Based on this, this paper further discusses the heterogeneous effects of CEO’s financial background on industrial AI transformation under different property rights. First of all, the credit allocation has an institutional bias towards state-owned enterprises, which is easy for state-owned enterprises to integrate more funds than their production and operation needs from the capital market (Wu et al., 2022), and a large amount of funds remain idle within enterprises, which makes state-owned enterprises generate strong demand for financial investment. Even if state-owned enterprises suffer losses due to financial investment, the government can still give them subsidies “from the bottom” so as to get them out of trouble, and their financial investment failure risk is small. Therefore, the CEO of state-owned enterprises has a large decision-making space. Secondly, private enterprises do not have the “unique” advantage of low external financing cost. Moreover, private enterprises face stronger external financing constraints and competitive pressure (Bai et al., 2021), and the motivation of holding financial assets has gradually shifted from the perspective of the traditional “profit-driven” to the “risk aversion” (Lashitew, 2017), which makes private enterprises less tolerant of transformation failure, and CEO decision-making becomes more cautious and conservative. To sum up, compared with private enterprises, CEOs with financial background of state-owned enterprises have a more “comfortable” financial investment environment, which makes it easier for them to flow funds to the financial sector, thus producing a “crowding out” effect on industrial AI transformation. Therefore, this paper expects that compared with private enterprises, CEO’s financial background has a stronger inhibitory effect on the industrial AI transformation of state-owned enterprises. The following econometric models are designed according to property rights nature of enterprise groups:


$$INM=c+\alpha_{1}FC+\overset{n}{\underset{j=1}{\sum }}\left(b_{j}Control\right)+\varepsilon \mathrm{(STATE = 1)}$$

$$INM=c+\alpha_{1}FC+\overset{n}{\underset{j=1}{\sum }}\left(b_{j}Control\right)+\varepsilon \mathrm{(STATE = 0)}$$

Among them, the nature of property rights (STATE) is a dummy variable, and listed companies belonging to state-owned enterprise groups are assigned to “1” and those belonging to private enterprise groups are assigned to “0.” Model (5) and model (6) are grouped based on the nature of ownership of enterprise groups. The regression results are shown in column (3) and column (4) of Table 6. It can be found that the regression coefficient of the CEO’s financial background does not pass the significance test in private companies and is significantly negative at the 5% level in state-owned companies. It indicates that compared with private enterprises, the inhibition effect of CEO’s financial background on industrial AI transformation is stronger in state-owned enterprises, which is consistent with the logic of the previous hypothesis.

### 6.3. Heterogeneity analysis of CEO’s financial background types

The experience of banking industry is a relatively special financial background (Gong et al., 2020), in order to further investigate the influence of CEO’s financial background on industrial AI transformation, this article divides CEO’s financial background into two dimensions: CEO’s banking financial background and CEO’s non-banking financial background. Compared with banks, other financial institutions have higher risks, higher work intensity and wider business scope, which makes CEOs with non-banking financial backgrounds better at identifying, selecting financial investment opportunities and accessing resources than those with banking backgrounds. Therefore, CEOs with non-bank financial background prefer financial investment, which in turn has a stronger inhibitory effect on the industrial AI transformation of manufacturing enterprises. Drawing on existing research (Mahmood-ur-Rahman, 2022), this paper establishes the following empirical model:


$$INM=c+\alpha_{1}BFC+\alpha_{2}NBFC+\overset{n}{\underset{j=1}{\sum }}\left(b_{j}Control\right)+\varepsilon$$

Among them, the CEO’s banking background (BFC) means that the CEO has only worked in banking financial institutions, that is, having banking financial background is assigned to 1, otherwise it is 0. The CEO’s non-banking financial background (NBFC) means that the CEO has only worked in non-banking financial institutions, that is, having a non-banking financial background is assigned a value of 1, otherwise it is 0. The results are shown in column (5) of Table 6, although the coefficients of banking financial background and non-banking financial background both show negative results, the estimated coefficient between the CEO’s non-banking financial background (NBFC) and industrial AI transformation is significantly negative at the level of 5%. It shows that compared with the CEO with only banking background, the CEO with only non-banking financial background are more able to inhibit industrial AI transformation, which is in line with the above hypothesis.

**Figure 3:**
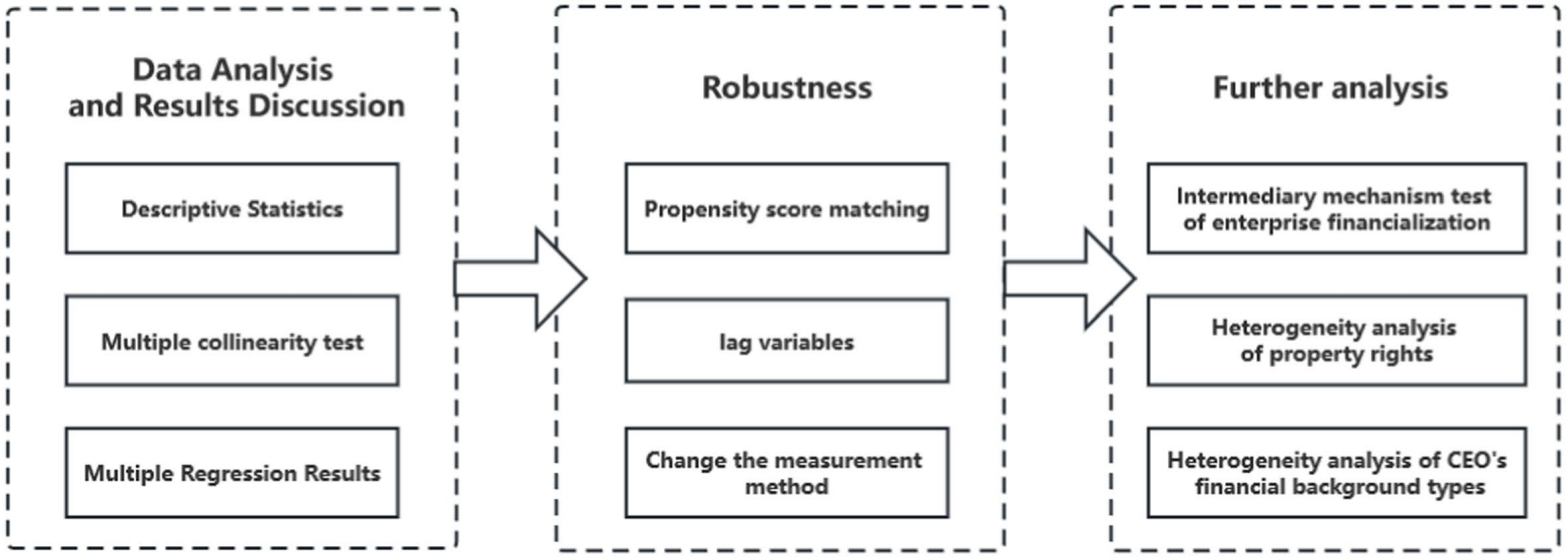
The process diagram of the methodology.

Accordingly, Figure 3 shows the process diagram of the methodology in this study.

## 7. Conclusion and implications

### 7.1. Conclusion

In the coming period, China’s development is still in an important strategic opportunity period, industrial AI transformation is receiving keen attention from the theoretical and practical circles, and the role of CEO with financial background is a hot topic in the society. This paper empirically tests the influence of CEO’s financial background on industrial AI transformation of manufacturing enterprises based on the Upper Echelons Theory and Imprinting Theory. The following conclusions are drawn: CEO’s financial background plays a significant role in inhibiting the industrial AI transformation of manufacturing enterprises, and when CEO works part-time in the parent company, it will strengthen the negative impact of CEO’s financial background on industrial AI transformation, which further clarifies the internal logic of the decision-making of industrial AI transformation of listed companies within the framework of enterprise groups. On this basis, this paper also tests the mechanism, and the results show that the CEO with financial background will promote enterprises financialization, thus inhibiting the transformation of industrial AI; Compared with private enterprise groups, the inhibiting effect of CEO financial background on industrial AI transformation is stronger in state-owned enterprise groups; After further distinguishing the banking and non-banking financial backgrounds, it is found that CEOs with only non-banking financial backgrounds are more able to inhibit industrial AI transformation.

### 7.2. Theoretical implications

Based on the above research findings, the following theoretical implications are obtained: First, this paper expands the research related to the influencing factors of industrial AI transformation based on Upper Echelons Theory and Imprinting Theory, clarifies the influencing mechanism of CEO financial background affecting industrial AI transformation from the perspective of managers’ behavior, and expands the application of Imprinting Theory. This paper adopts a manual collection method to organize industrial AI transformation data, responding to the call to explore the relationship between AI and strategic transformation of manufacturing companies (Burstrm et al., 2021), and provides a theoretical basis for CEO to establish correct psychological cognition and Investment concept. Second, our study further enriches the research framework on the economic consequences of CEO financial background. Previous studies have mainly explored the effects of CEO financial backgrounds on internal control weaknesses, earnings management, timeliness of audit reports and corporate innovation (Gounopoulos and Pham, 2018; Salehi et al., 2018; Oradi et al., 2020; Yang et al., 2021). The findings of this paper provide theoretical support and new solution ideas at the micro level for re-examining and solving problems such as the chaos of financial asset investment and excessive financialization of enterprises in the process of industrial AI transformation in China’s manufacturing. Third, taking the part-time job of the subsidiary CEO in the parent company as the entry point, this paper is not limited to a single enterprise but involves the management practice of resource allocation within the enterprise groups, providing theoretical reference for the scientific design of the governance mechanism of parent-subsidiary companies and the prevention and resolution of systemic financial risks.

### 7.3. Practical implications

The study’s practical implications are discussed below. First, CEOs should be alert to the negative impact that may be brought by the imprinting effect, clearly recognize and prudently treat financial investment experience and information processing ability advantages, and appropriately allocate financial assets based on business conditions of the enterprise and the maturity of their AI applications. Moreover, CEOs need to establish awareness of financial risk prevention and enhance their comprehensive quality and professional capabilities. Especially for state-owned enterprises and CEOs with non-banking financial background, they can self-evaluate their own behavior according to relevant evaluation indicators, correct their cognitive and psychological biases in time, and make reasonable investment decisions.

Second, enterprises should pay attention to the diversity of the working backgrounds of the executive team members when selecting executives, appropriately control the proportion of executives with financial background in the decision-making level to build a more diversified management, so as to maximize the positive effect of the imprinting mechanism and control the degree of financialization of enterprise within a reasonable range. In addition, enterprises should improve the restraint and incentive mechanisms on CEO decision-making power, and strengthen the supervision and restraint on CEO by using internal and external corporate governance mechanisms such as majority shareholder governance, board governance, and third-party risk assessment institutions on the premise of not damaging the work enthusiasm, that is, weakening the voice of CEOs with financial backgrounds, especially CEOs with non-banking financial background, in corporate financial investment decisions, so as to avoid excessive financialization of enterprises caused by CEOs’ original career tendencies, which would hinder or even destroy industrial AI transformation.

Third, government departments should strengthen capital market governance, improve the lending function of financial markets, and reduce the external financing costs of enterprises (Hussain H. et al., 2021; Wen et al., 2022), improve the functional attributes of financial markets and financial assets so that finance can return to the source of serving the real economy. Moreover, the government should eliminate the institutional credit financing discrimination brought by the form of ownership and strengthen the supervision over the allocation of financial assets by state-owned enterprises. Considering that excessive financialization will reduce the efficiency of real investment and efficient firms may not fear negative signaling effects (Hussain R. Y. et al., 2021), the government should establish a dynamic monitoring mechanism for enterprise financialization and scientifically set the regulatory threshold for enterprise financialization, and once the monitoring detects that the weight of enterprise financial asset allocation exceeds the regulatory threshold, severe punishment will be imposed on enterprises with excessive financialization, so as to restrict enterprise financialization.

### 7.4. Limitations and future directions

Several limitations should be noted and addressed in future research. First, the examination of the impact mechanism in this paper is limited to the mediating path of enterprise financialization, and recent studies have shown that debt maturity decisions are more crucial than capital structure decisions in venture capital (Wen et al., 2021; Hussain et al., 2022), so future studies can comprehensively understand the mechanism of CEO’s financial background on industrial AI transformation by examining other mediating variables such as debt maturity. Second, this paper only considers the property rights as a micro feature of an enterprise in the heterogeneity analysis. Yang and Shen (2022) analyze the influence of the external macro-features of an enterprise from the aspects of monetary policy, industry competition, and institutional environment. Subsequent studies can examine the influence of factors such as CEO’s working time and position hierarchy in financial institutions on industrial AI transformation based on an in-depth examination of the internal and external governance contexts of firms.

## Data availability statement

The original contributions presented in the study are included in the article/supplementary material, further inquiries can be directed to the corresponding author.

## Author contributions

All authors listed have made a substantial, direct, and intellectual contribution to the work and approved it for publication.

## Funding

This research was funded by the National Natural Science Foundation of China (grant number 71972117), Shandong Provincial Natural Science Foundation, China (grant number ZR2022MG028), Taishan Scholar Foundation of Shandong Province (grant number tsqn202103095), and A Project of Shandong Province Higher Educational Science and Technology Program (grant number 2021RW009).

## Conflict of interest

The authors declare that the research was conducted in the absence of any commercial or financial relationships that could be construed as a potential conflict of interest.

## Publisher’s note

All claims expressed in this article are solely those of the authors and do not necessarily represent those of their affiliated organizations, or those of the publisher, the editors and the reviewers. Any product that may be evaluated in this article, or claim that may be made by its manufacturer, is not guaranteed or endorsed by the publisher.
